# Supplementary material for: Genetically Encoded Fluorescent Sensor for Poly-ADP-Ribose
Source: Int J Mol Sci. 2020 Jul 15;21(14):5004. doi: 10.3390/ijms21145004 (PMC7404130; doi:10.3390/ijms21145004)
Supplement: Supplementary file 1 [file ijms-21-05004-s001.pdf]

Genetically encoded fluorescent sensor for poly-ADP-ribose

Ekaterina O. Serebrovskaya, Nadezda M. Podvalnaya, Varvara V. Dudenkova, Anna S. Efremova, Nadya G. Gurskaya, Dmitry A. Gorbachev, Artem V. Luzhin, Omar L. Kantidze, Elena V. Zagaynova, Stanislav I. Shram, Konstantin A. Lukyanov

Supplementary Materials

Figure S1	Protein sequence of Turquoise2-WWE and Venus-WWE.
Figure S2	Nucleus-to-cytoplasm distribution of FP-WWE constructs and FRET efficiency change after H_2O_2 treatment.
Figure S3	sPARroW response to PAR accumulation inducing stimulus in U2OS stable cell line.
Figure S4	sPARroW response in transiently transfected U2OS cells after H_2O_2 treatment.
Figure S5	Immunofluorescence analysis of PAR accumulation in U2OS cells.

Turquoise2-WWE

MVSKGEELFTGVVPILVELDGDVNGHKFSVSGEGEGDATYGKLTLKFICTTGKLPVPWPTLVTT
LSWGVQCFARYPDHMKQHDFFKSAMPEGYVQERTIFFKDDGNYKTRAEVKFEGDTLVNRIELKG
IDFKEDGNILGHKLEYNYFSDNVYITADKQKNGIKANFKIRHNIEDGGVQLADHYQQNTPIGDG
PVLLPDNHYLSTQSALSKDPNEKRDHMVLLEFVTAAGITLGMDELYKGGGSSGGGQISYASREF
NGEYAWYYEGRNGWWQYDERTSRELEDAFSKGKKNTEMLIAGFPYVADLENMVQYRRNEHGRRR
KIKRDIIDIPKKGVAGLRLDC

Venus-WWE

MVSKGEELFTGVVPILVELDGDVNGHKFSVSGEGEGDATYGKLTLKLICTTGKLPVPWPTLVTT
LGYGLQCFARYPDHMKQHDFFKSAMPEGYVQERTIFFKDDGNYKTRAEVKFEGDTLVNRIELKG
IDFKEDGNILGHKLEYNYNSHNVYITADKQKNGIKANFKIRHNIEDGGVQLADHYQQNTPIGDG
PVLLPDNHYLSYQSALSKDPNEKRDHMVLLEFVTAAGITLGMDELYKGGGSSGGGQISYASREF
NGEYAWYYEGRNGWWQYDERTSRELEDAFSKGKKNTEMLIAGFLYVADLENMVQYRRNEHGRRR
KIKRDIIDIPKKGVAGLRLDC

Fig. S1. Protein sequence of Turquoise2-WWE and Venus-WWE. Sequences of Turquoise2, Venus, and WWE are highlighted in cyan, green and pink, respectively. Position of Arg163 mutated in the control constructs to abolish PAR binding (R163A) is highlighted in red.

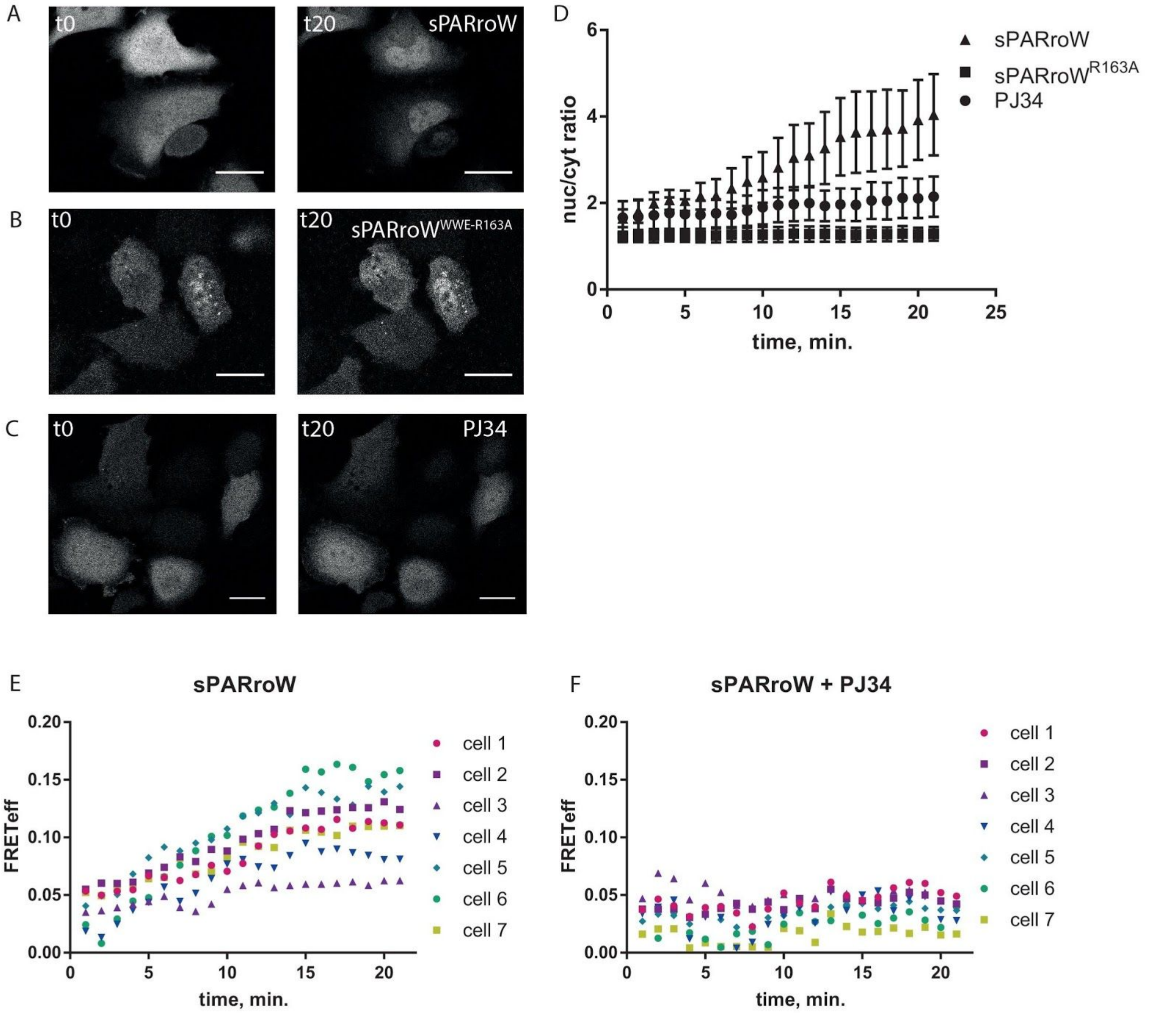

Fig. S2. Nucleus-to-cytoplasm distribution of WWE-FP constructs and FRET efficiency change after H_2O_2 treatment. (A-C) Confocal microscopy images of cells in the donor channel before (left) and 20 min after (right) addition of H_2O_2. Representative images of cells expressing sPARroW (A), sPARroW-R163A (B) or sPARroW + PJ34 (C) are shown. Scale bars 25 μm. (D) Nuclear-to-cytoplasm ratio change after H_2O_2 treatment. (E) Single-cell measurement of FRET efficiency change after H_2O_2 exposure. (F) Single-cell measurement of FRET efficiency change in PJ34 pretreated cells after H_2O_2 exposure.

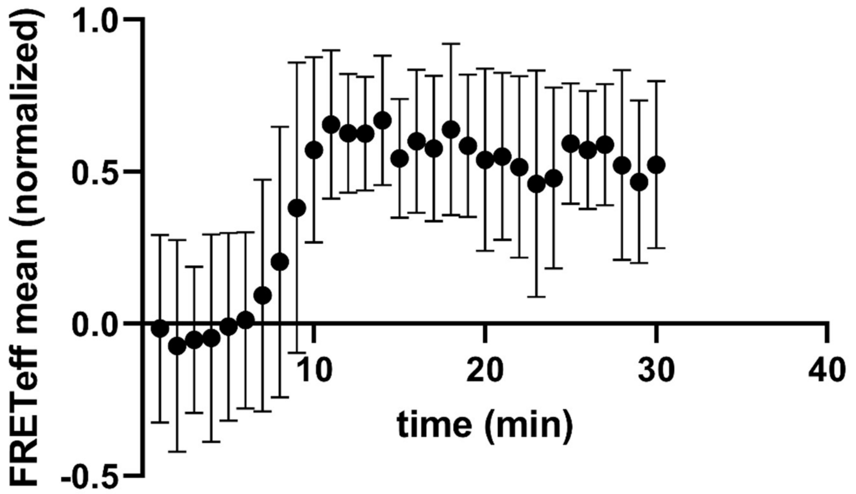

Fig. S3. sPARroW response to PAR accumulation inducing stimulus in U2OS stable cell line. Mean fluorescence intensity signals from donor, acceptor and FRET channels were used to calculate FRET efficiency in selected regions of interest according to the formula in Methods section. Mean values ±SD are depicted.

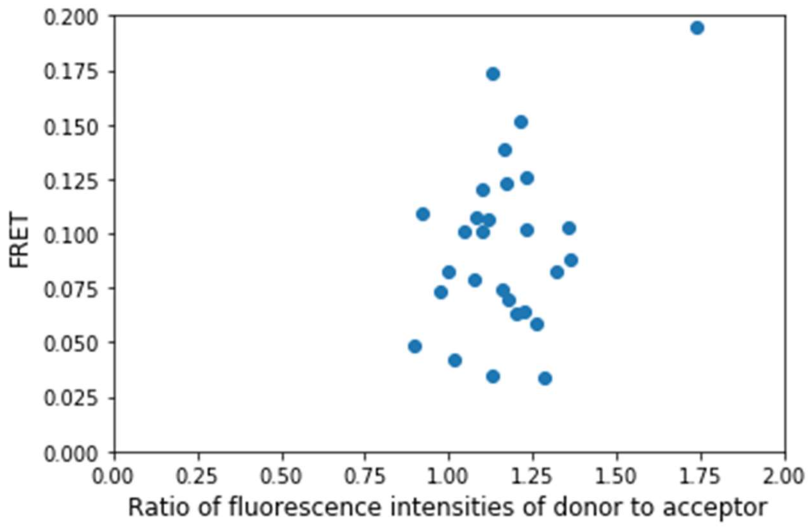

Fig. S4. sPARroW response in transiently transfected U2OS cells after H_2O_2 treatment. Cell were transiently transfected with Turquoise2-WWE and Venus-WWE encoding plasmids at 1:1 ratio. Graph shows dependence of FRET efficiency on ratio of fluorescence signals of the donor to the acceptor (each dot corresponds to an individual cell). Note strong cell-to-cell variability of FRET efficiency independent on the donor/acceptor ratio within the observed narrow range.

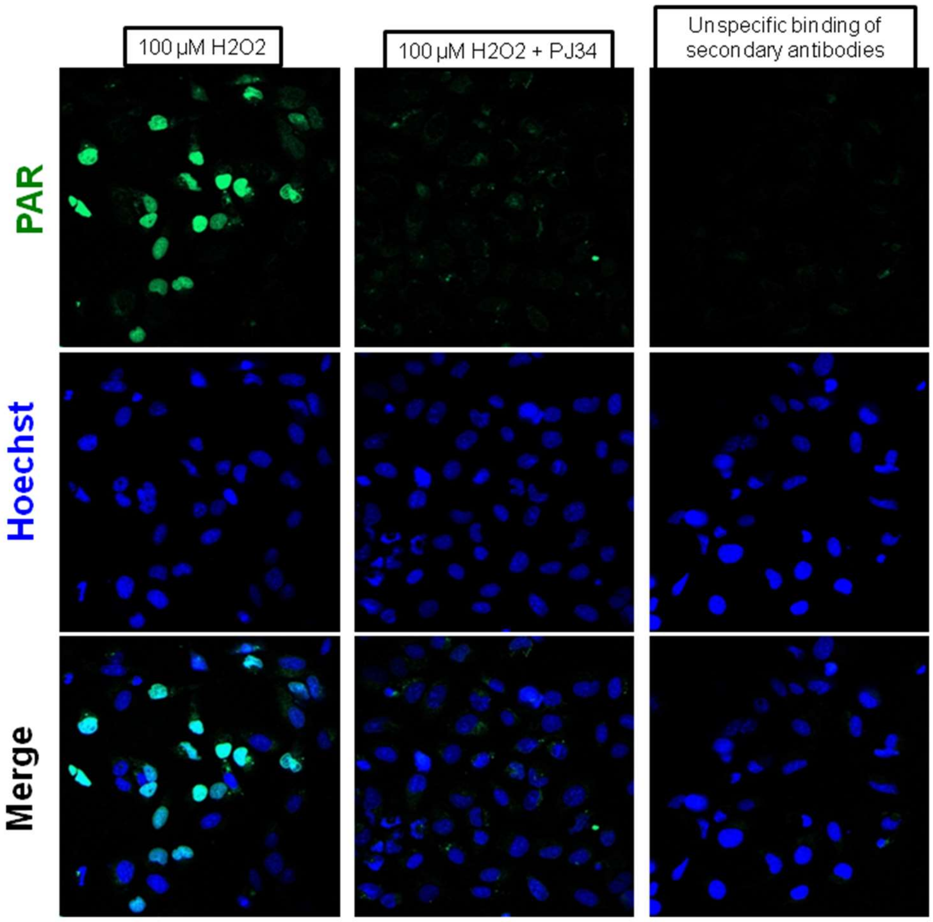

Fig. S5. Immunofluorescence analysis of PAR accumulation in U2OS cells. PAR-specific primary antibodies and Alexa Fluor 488-labeled secondary antibodies were used. Left column – cells treated with H_2O_2 for 20 min. Middle column – cells pretreated with PARP inhibitor PJ34 and treated with H_2O_2 for 20 min. Right column – control cells with no staining by the PAR-specific primary antibodies. Upper row – green fluorescence of the secondary antibodies; middle row – blue fluorescence of nuclei stained with Hoechst; bottom row – overlay of the two channels. Corresponding images were taken under the same conditions. Note strong cell-to-cell variability of PAR accumulation.
